# *In vivo* GluCEST MRI: Reproducibility, background contribution and source of glutamate changes in the MPTP model of Parkinson’s disease

**DOI:** 10.1038/s41598-018-21035-3

**Published:** 2018-02-13

**Authors:** Puneet Bagga, Stephen Pickup, Rachelle Crescenzi, Daniel Martinez, Arijitt Borthakur, Kevin D’Aquilla, Anup Singh, Gaurav Verma, John A. Detre, Joel Greenberg, Hari Hariharan, Ravinder Reddy

**Affiliations:** 1 Center for Magnetic Resonance and Optical Imaging, Department of Radiology, University of Pennsylvania, Philadelphia, PA United States; 2 Department of Pathology and Laboratory Medicine, Children’s Hospital of Philadelphia, Philadelphia, Pennsylvania USA; 3 Department of Neurology, University of Pennsylvania, Philadelphia, PA United States; 4 Centre for Biomedical Engineering, Indian institute of Technology, New Delhi, India

## Abstract

Glutamate Chemical Exchange Saturation Transfer (GluCEST) MRI is a recently developed technique to image glutamate. In the present study, we evaluated the reproducibility and background contamination to the GluCEST and source of the GluCEST changes in a mouse model of Parkinson’s disease. Repeated measurements in five mice demonstrated an intra-animal coefficient of variation (CV) of GluCEST signal to be 2.3 ± 1.3% and inter-animal CV of GluCEST to be 3.3 ± 0.3%. Mice were treated with MPTP to create a localized striatal elevation of glutamate. We found an elevation in the GluCEST contrast of the striatum following MPTP treatment (Control: 23.3 ± 0.8%, n = 16; MPTP: 26.2 ± 0.8%, n = 19; p ≤ 0.001). Additionally, the positive association between glutamate concentration measured via ^1^H MRS and GluCEST signal was used to estimate background contribution to the measured GluCEST. The contribution of signal from non-glutamate sources was found to be ~28% of the total GluCEST. Immunohistochemical analysis of the brain showed co-localization of glutamate with GFAP in the striatum. This suggests that the elevated glutamate present in the striatum in this mouse model reflects astroglial proliferation or reactivity due to the action of MPTP. The potential of GluCEST as a biomarker for imaging inflammation mediated gliosis is discussed.

## Introduction

Glutamate Chemical Exchange Saturation Transfer (GluCEST) MRI is a recently developed technique to image parenchymal glutamate in brain^1,2^. GluCEST provides indirect detection of glutamate *in vivo* by measuring the exchange of glutamate amine protons with bulk water, and provides greater sensitivity and spatial resolution than conventional ^1^H magnetic resonance spectroscopy (^1^H MRS) techniques. GluCEST MRI has been used to study neurodegeneration due to amyloid pathology^3^, tau pathology induced synapse loss^4,5^, human epilepsy^6^, Huntington’s disease^7^, schizophrenia^8^, MPTP induced parkinsonism^9^ and to map changes in glutamate distribution in the brain. However, because the GluCEST method relies on indirect detection, it is potentially contaminated by contributions of other metabolites exhibiting proton chemical exchange^1^.

In a previous report, we demonstrated an elevation of striatal glutamate after 1-methyl-4-phenyl-1,2,3,6-tetrahydropyridine (MPTP) treatment in mice using both GluCEST MRI and ^1^H MRS^9^. MPTP is a neurotoxin which selectively kills the dopaminergic neurons in the substantia nigra pars compacta and striatum^10,11^. Leveraging the spatial resolution of GluCEST, we found that striatal and motor cortex GluCEST signal changes were inversely correlated with motor function. A strong correlation between elevated glutamate and histologically determined glial reactivity was also observed, suggesting the possibility that the observed glutamate increase was predominantly derived from astrocytes. However, the previous study did not assess the repeatability of GluCEST MRI and potential contribution of other non-glutamate sources to the *in vivo* GluCEST signal.

The current study focuses on the reliable repeatability and specificity of GluCEST as a measure of brain glutamate. By correlating the GluCEST signal with ^1^H MRS derived glutamate concentration measured in the MPTP treated and control mice, we estimated the background non-glutamate contribution to the total GluCEST contrast. In addition, immunohistochemical analysis including small molecule glutamate staining was performed to assess the origin of elevated glutamate in this model.

## Results

### Reproducibility of GluCEST MRI

**Figure 1:**
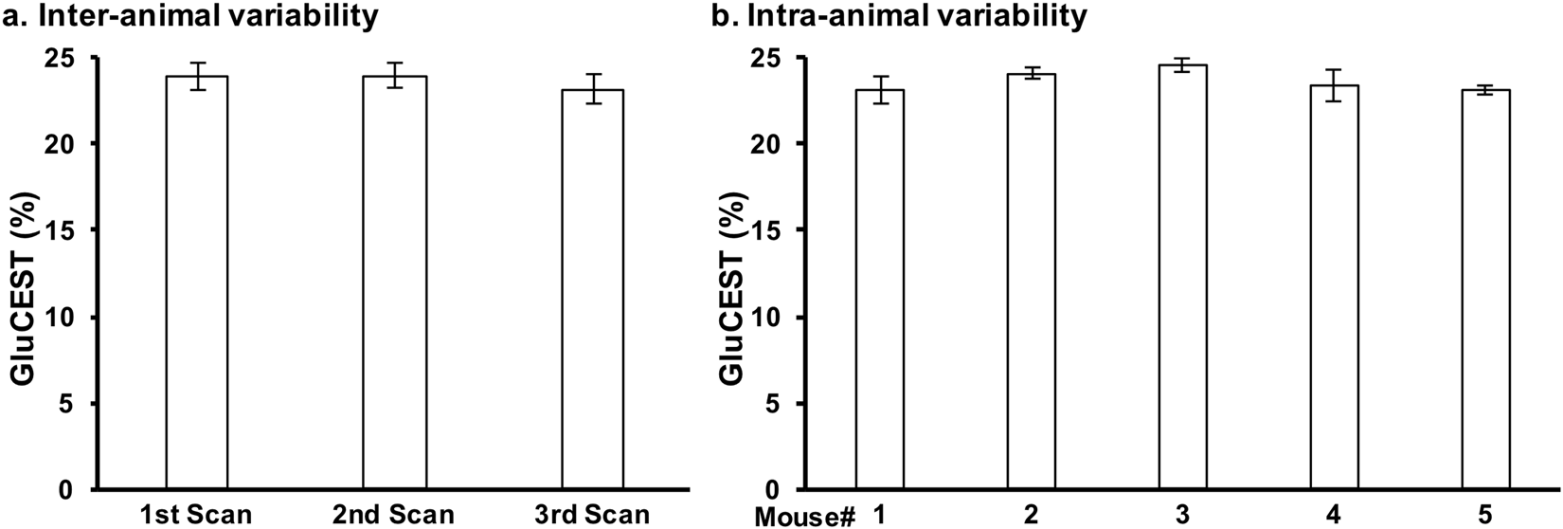
Inter- and intra-animal variability of the GluCEST MRI. Five mice were scanned at three timepoints, with second and third scan at 3^rd^ and 6^th^ day following the first scan. The GluCEST values were calculated from ROIs placed in the striatum of the brain on the MR slice. (**a**) Average of GluCEST contrast from the striatum of the 5 mice from each scan and (**b**) Average of GluCEST contrast from 3 scans in each mouse. Data are presented as Mean ± SD.

GluCEST contrast from the striatum of five mice scanned thrice with a minimum two-day rest between scans was measured. The average coefficient of variation (CV) for the intra-animal variability was found to be 2.3 ± 1.3%. Additionally, the CV for the inter-animal variability was found to be 3.3 ± 0.3% (Fig. 1b). This demonstrates the high degree of repeatability and low inter-animal variability of GluCEST scans performed in mice.

### GluCEST MRI of striatum following MPTP treatment

**Figure 2:**
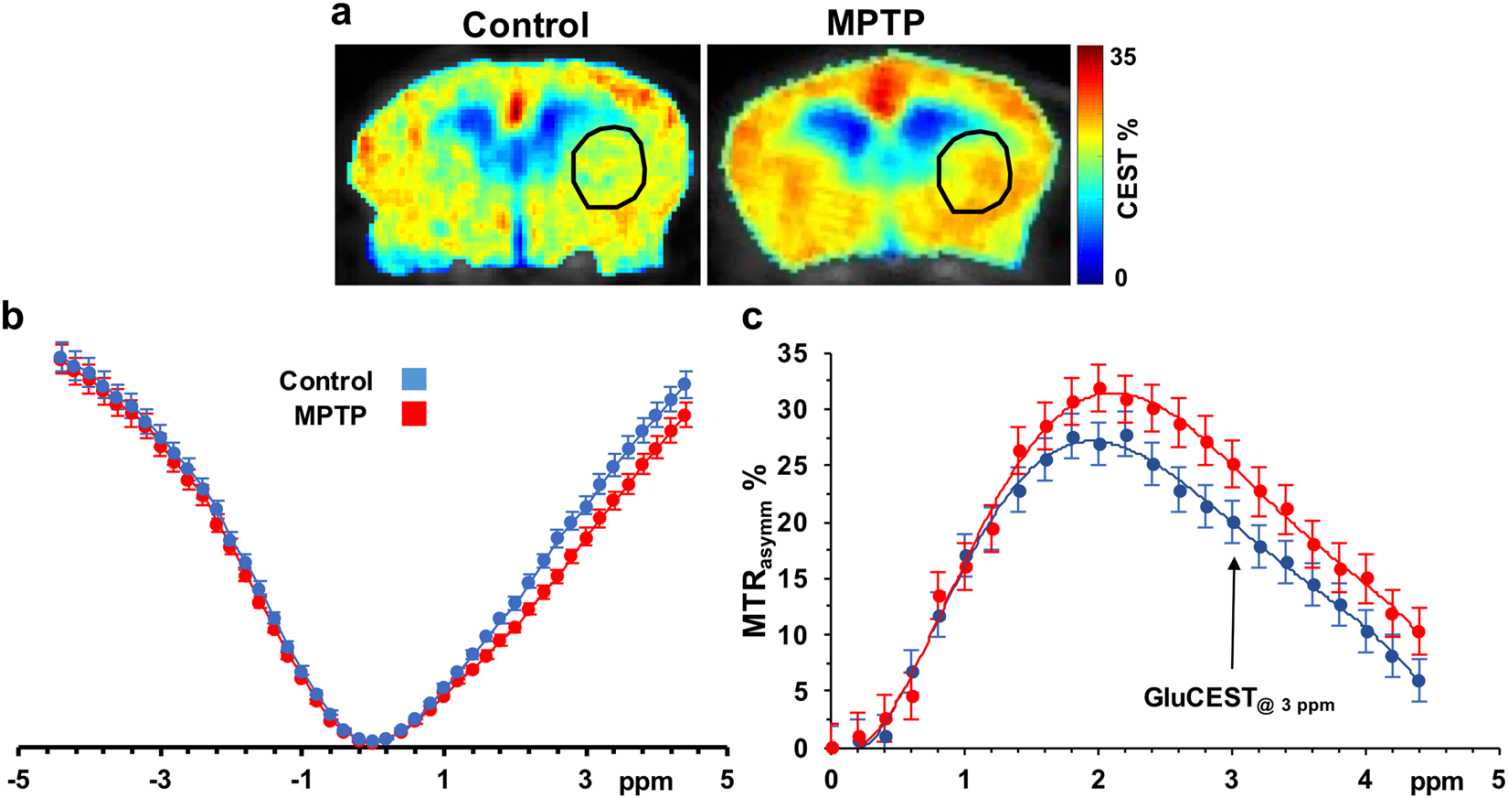
GluCEST asymmetry from the striatum. (**a**) MTR_asymm_ map at 3 ppm for GluCEST scans of control mouse brain (left) and MPTP treated mouse brain (right). (**b**) Z-spectra and (**c**) Asymmetry plot generated from the ROI’s placed in the striatum of mice of the two groups (control, n = 3, blue; MPTP, n = 3, red) showing a difference between the asymmetry at 3 ppm.

A MTR_asymm_ plot from the ROI placed in the striatum was generated by acquiring CEST images at frequency offsets 0 to ±5 ppm with step size 0.2 ppm. GluCEST contrast was measured as the asymmetry between an image obtained with saturation at the resonant frequency of exchangeable amine protons (+3 ppm), and an image with saturation equidistant upfield from water (−3 ppm) (Eq. 1). MTR_asymm_ plots show a difference between the control and MPTP mice at 3 ppm indicating that the glutamate elevation is the primary cause for the difference between the two groups (Fig. 2).

### ^1^H MRS from the striatum

**Figure 3:**
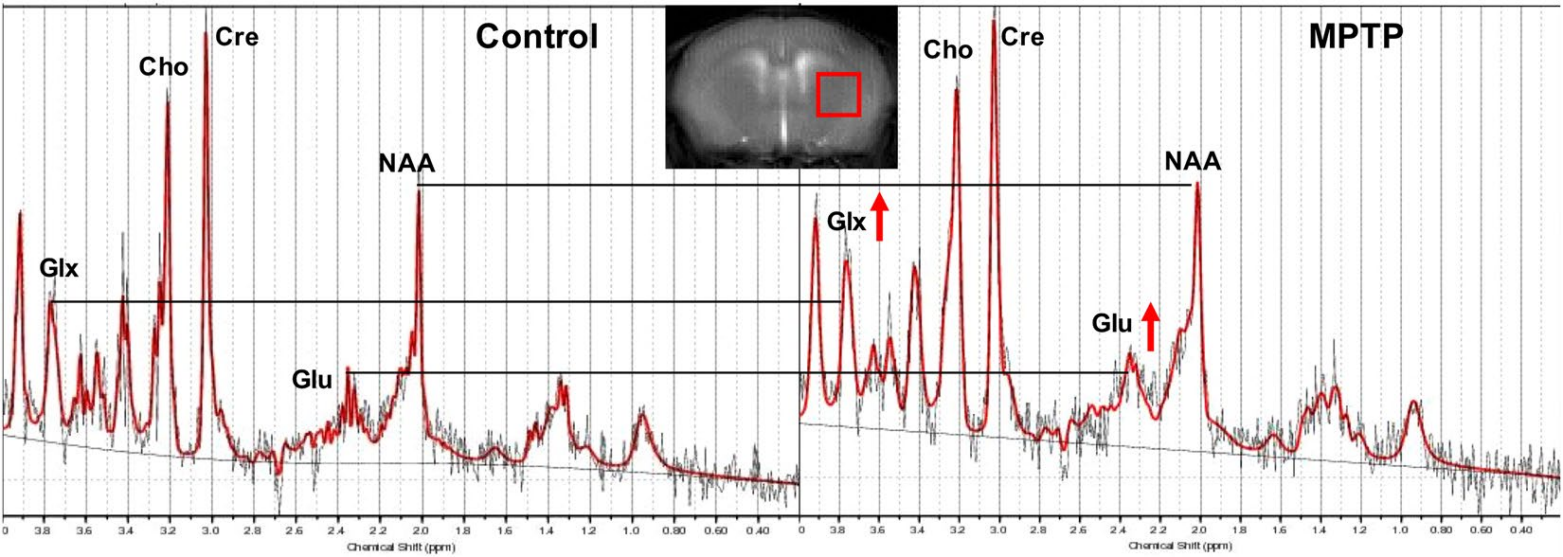
^1^H MRS of the striatum of mice. ^1^H MR spectra were acquired from a 2 × 2 × 2 mm^3^ voxel placed in the striatum (as shown in the inset). Raw spectra were processed using the LCmodel. Raw spectra (black) and fitted spectra (red) are shown with increased glutamate observed following exposure to MPTP. Other MRS observable metabolites were unaltered. Glu Glutamate, Cre total creatine, NAA N-Acetylaspartate, Cho Choline, Glx Gutamte + Glutamine.

^1^H MRS was performed in a voxel placed in the striatum of the mice in both the groups (Control n = 16, MPTP n = 14; Fig. 3). The LCmodel analysis of the spectra from control and MPTP group showed that the glutamate was elevated in the MPTP treated group (Fig. 3). The other metabolites were found to be unaltered appreciably in the MPTP group as compared to the control.

### Elevation of striatal GluCEST contrast and [Glu] in MPTP group

**Figure 4:**
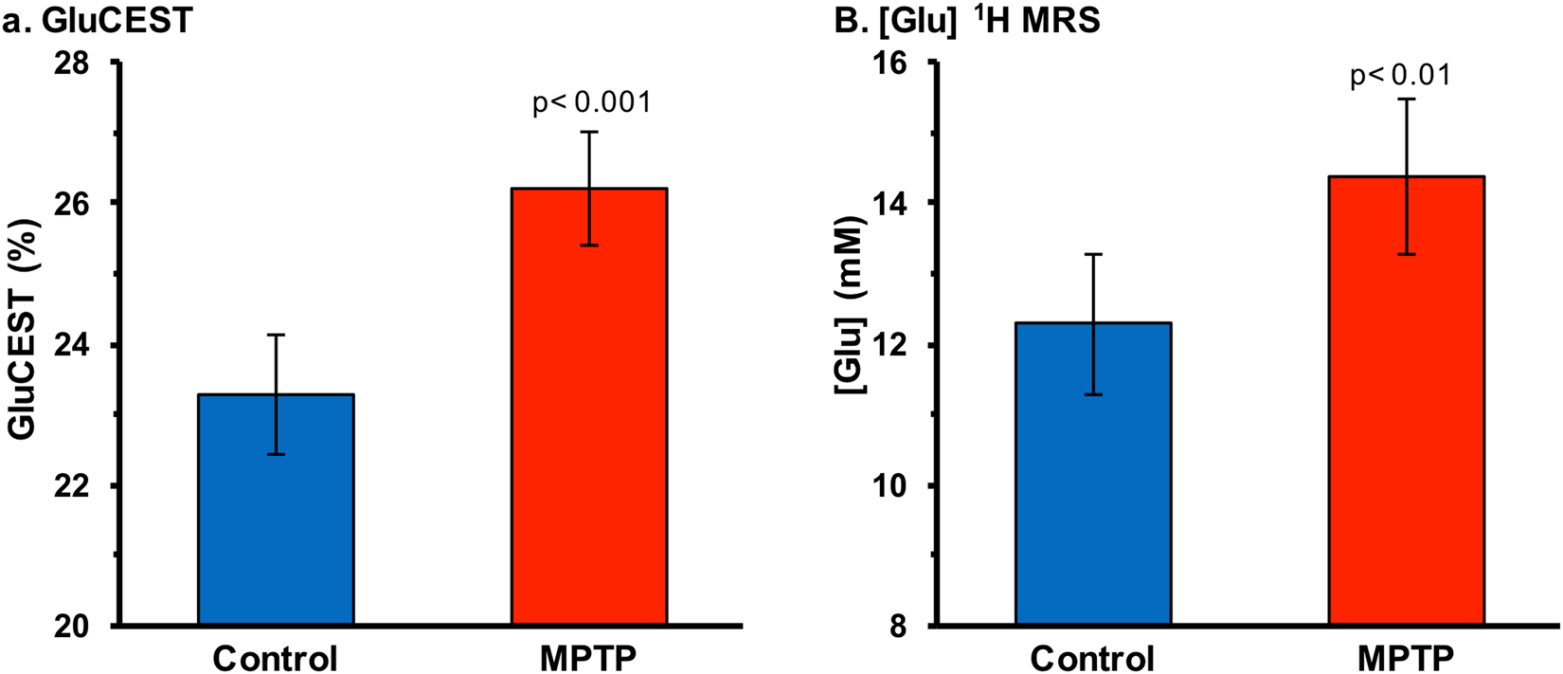
GluCEST MRI and ^1^H MRS in the striatum. (**a**) GluCEST contrast was found to be higher in the striatum of the MPTP group (26.2 ± 0.8%, n = 19) compared to the control group (23.3 ± 1.0%, n = 16; p ≤ 0.001). (**b**) Glutamate measured via ^1^H MRS in the striatum was also found to be higher in the MPTP group (control: 12.3 ± 1.0 mM, n = 16; MPTP: 14.4 ± 1.1 mM, n = 19; p ≤ 0.005).

**Figure 5:**
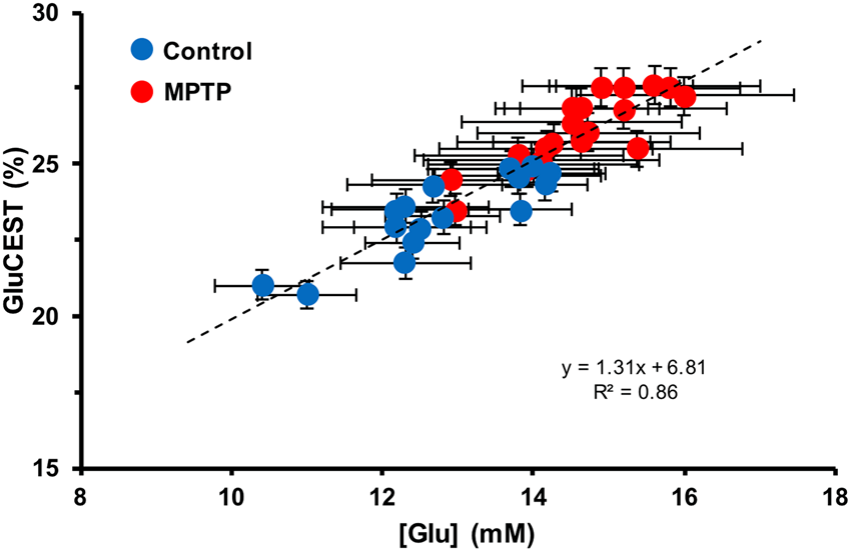
Correlation between GluCEST contrast and ^1^H MRS. Plot showing the mean values of GluCEST contrast and glutamate concentration in the striatum for the control and MPTP treated mice. All data are presented as Mean ± SD.

GluCEST imaging showed elevated glutamate-dependent signal changes in the striatum of MPTP mice as compared to control mice (control: 23.3 ± 0.8%, n = 16; MPTP: 26.2 ± 0.8%, n = 19; p ≤ 0.001, Fig. 4a). Figure 3b shows the glutamate concentration measured from ^1^H MRS spectra from the striatum for both the groups. Compared to control mice, ^1^H MRS derived glutamate was found to be elevated in the MPTP mice (control: 12.3 ± 1.0 mM, n = 16; MPTP: 14.4 ± 1.1 mM, n = 19; p ≤ 0.01, Fig. 4b). Figure 5 shows the mean GluCEST contrast and the glutamate concentration measured via ^1^H MRS of the two groups of mice.

The bivariate fit on the data was performed using 3 models to fit GluCEST with [Glu] and sample type with a p < 0.01 as significant (Supplementary material). Analysis of variance showed that the most parsimonious model was sufficient (p < 0.01) to regress GluCEST with [Glu] where GluCEST (%) = 1.31 (%/mM) × [Glu] (mM) + 6.81% with R^2^ = 0.86 (Fig. 5). Furthermore, we observed that residuals were indeed normally distributed with this fit. The intercept of 6.81% could be attributed to a background contribution from non-glutamate sources (e.g. creatine and other amine/amide proton containing molecules).

### Increased striatal glial reactivity and glutamate following MPTP treatment

**Figure 6:**
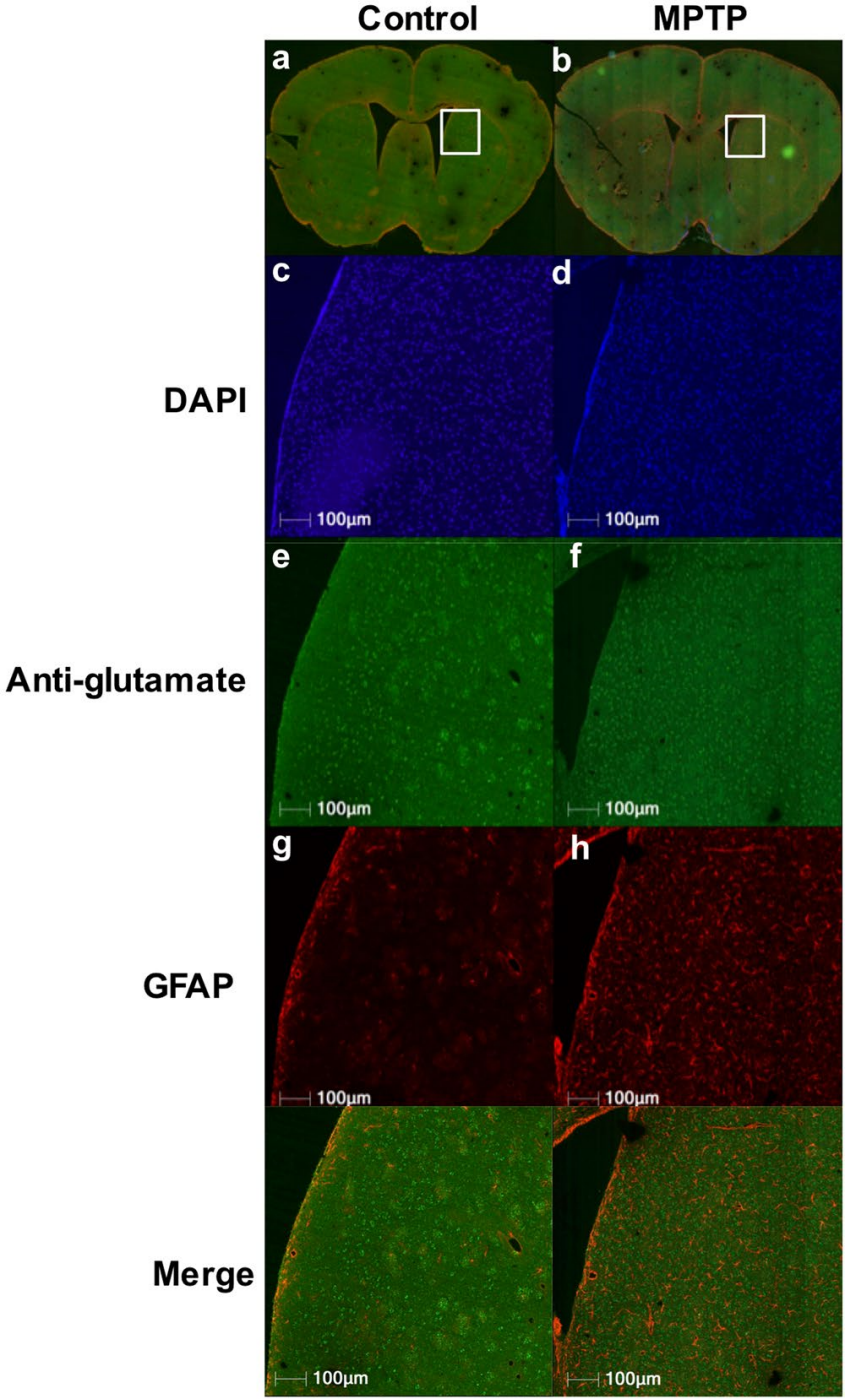
Immunohistochemical analysis of brain following MPTP exposure. (**a,b)** Representative sections of brain from the two groups of mice control (**a**) and MPTP (**b**) showing the striatum region outlined (white square) where the analysis was performed. (**c,d)** DAPI staining in the MPTP (**d**) compared to control (**c**) mice. (**e,f)** Anti-glutamate stain showing higher glutamate in the MPTP (**f**) treated compared to control (**e**) mice. Additionally, glutamate staining was localized in clusters in the control mice indicating contrast consistent with neuronal localization, whereas diffused glutamate staining in the MPTP is consistent with localization to glial axons in the neuropil. (**g,h)** GFAP staining showing dramatically high astrocytic reactivity in the MPTP treated striatum. (**i**) Merge of anti-glutamate, GFAP and DAPI showing the GFAP and glutamate staining in the MPTP striatum.

Immunohistochemical analysis was performed on the fixed slices containing the striatum in the control and MPTP group (Fig. 6a,b). A small region in the striatum was imaged further for qualitative analysis. DAPI nuclei staining showed no change in the MPTP striatum (Fig. 6c,d). Anti-glutamate staining was found to be higher in the MPTP group compared to the control (Fig. 6e,f). Additionally, the glutamate stain was more diffused in the MPTP striatum compared to the control. This may be due to the presence of glutamate in cell bodies other than the neurons. GFAP reactivity was drastically higher in the MPTP group suggesting reactive astrocytosis (Fig. 6g,h). The image showing merge of DAPI, GFAP and anti-glutamate stains showed the presence of glutamate in neurons as well as reactive astrocytes in the striatum of an MPTP treated mouse (Fig. 6i).

### Co-localization of glutamate and reactive glia in MPTP treated mouse

**Figure 7:**
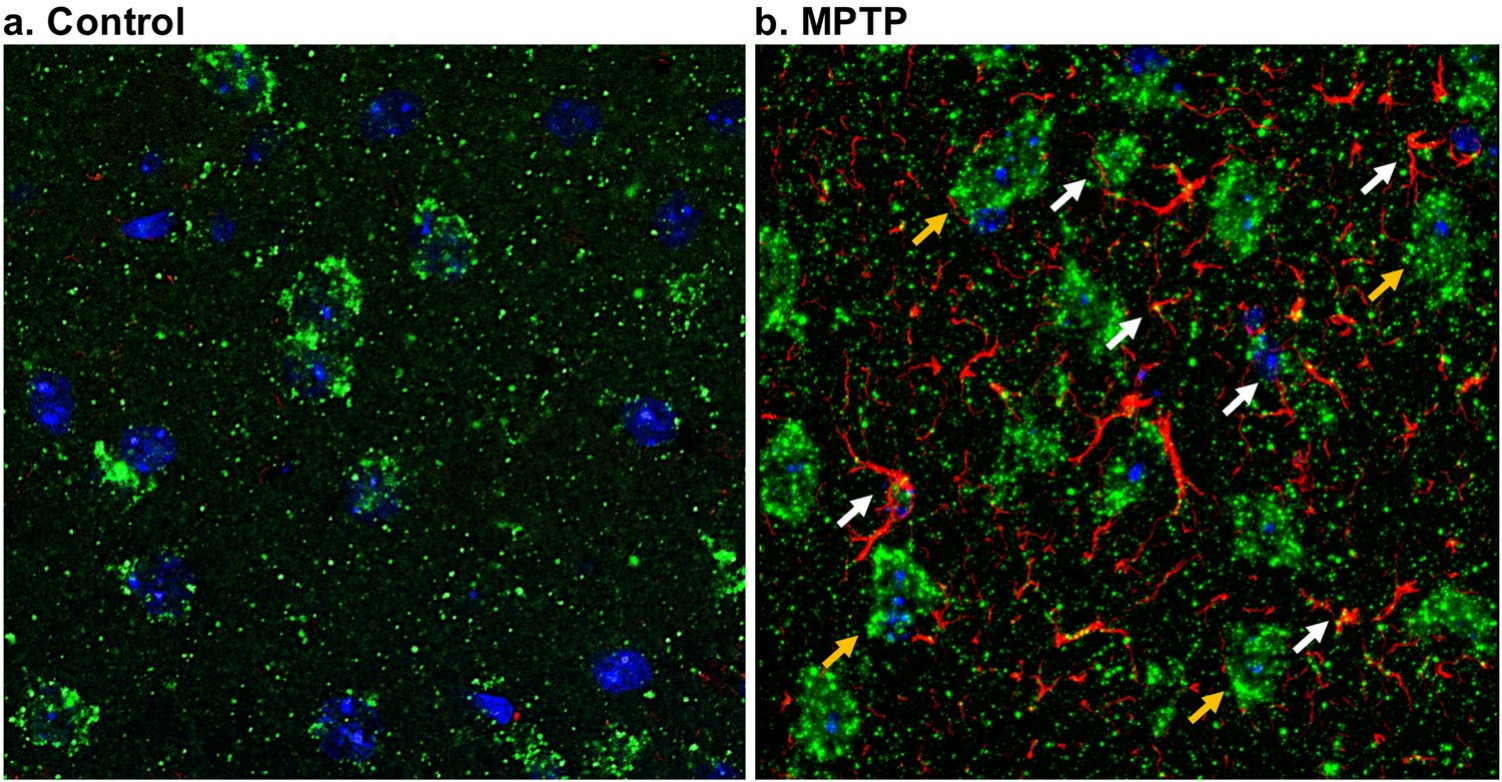
Co-localization of glutamate and GFAP. The confocal microscopy of the brain slices were performed in a 63 × zoomed region in the striatum of the control (**a**) and MPTP treated mouse (**b**). The figure shows the cell bodies with nuclear stain DAPI (blue) having glutamate (green) and GFAP (red) immunostaining. Neuronal nuclei are bigger and are found to be co-localized with neuronal glutamate (yellow arrows), whereas, astrocytic glutamate appeared to co-localize with GFAP staining. Areas marked with white arrows show the co-localization of glutamate and GFAP in the reactive glia.

Confocal microscopy was used to detect the co-localization of the glutamate and GFAP. Figure 7 shows the merged image of anti-glutamate and GFAP stains in the striatum of control (Fig. 7a) and MPTP treated mice (Fig. 7b). While most of the glutamate was found to be around the neuronal nuclei, a few striations in the reactive astrocytes were found to be co-localized with glutamate in the highly-zoomed area in striatum of MPTP treated mice (white arrows). This indicates that elevated glutamate may be present in the reactive glial cells followed by the MPTP action.

## Discussion

Reproducible GluCEST MR imaging is vital to detect subtle changes in glutamate levels *in vivo* under pathological conditions. We observed low CV of GluCEST contrast in repeated scans of the same animal, compared to the level of group differences in GluCEST between control and MPTP treated mice, indicating high degree of reproducibility of the GluCEST maps. Inter-animal variability of GluCEST among the age matched animals was also low. The high reproducibility of the results in the GluCEST scans enabled reliable measurement of the local changes in the glutamate levels caused by the MPTP toxicity to the striatal dopaminergic neurons.

Asymmetry analysis of brain GluCEST measured by acquiring the CEST weighted images throughout the z-spectrum confirmed that the change in the MTR_asymm_ was primarily due to amine protons, which are present on glutamate and other molecules. Creatine is another amine proton containing molecule present in relatively high concentration in brain parenchyma^12–14^. However, the ^1^H MRS analysis showed no change in striatal creatine level following MPTP treatment, and there were no detectable changes in other metabolites measured via ^1^H MRS. MT changes may contribute to the changes in the GluCEST. However, the saturation parameters used in the study were optimized to enhance the contribution of glutamate to the CEST_asymm_ at 3 ppm. Our earlier published work estimated theoretically that ~73% of GluCEST contrast is attributable to glutamate with these parameters, while Gln was shown to not contribute to GluCEST^1^. In addition, the relayed Nuclear Overhauser Effect (rNOE) contribution to the CEST is negligible at the parameters used for GluCEST acquisition^15,16^. These findings indicate that GluCEST contrast changes in the MPTP treatment model are primarily due to glutamate. Glutamate has been shown to be elevated in the striatum of mice exposed to MPTP using ^1^H MRS previously^17,18^. In this study, we found that the other metabolites were unaltered following the MPTP exposure. We treated the mice for 7 days and we performed the scanning after a one day delay. Most of the earlier studies which show changes in other metabolites in rodent as well as primate brains have a significant amount of delay (>2 weeks) between the treatment and the metabolic measurements^18–20^. This may be one of the reasons that in this study, there were no changes in other metabolites.

The experimentally measured glutamate concentration derived from ^1^H MRS and GluCEST contrast changes were used to evaluate the background contributions to the total GluCEST signal. The y-intercept in the Fig. 5 indicates the GluCEST background when the concentration of cerebral glutamate is zero. This showed that the GluCEST contrast in the brain still exists even when there is no glutamate. The remaining GluCEST values are coming from the glutamate and hence the contribution of glutamate to GluCEST was evaluated using the background information as shown in equation 2. We found experimentally here that the contribution of glutamate to the total GluCEST signal is 72%, implying that the remaining 28% signal may arise due to other molecules. These results are consistent with our previous theoretical estimates that the background contributions to GluCEST from non-glutamate sources is 25–30%^1^. The background contribution likely derives from molecules with amine/amide groups that are not readily observed on ^1^H MRS, perhaps large molecules with very short T_2_ relaxation times. Future studies are required to further characterize contributions to GluCEST from non-glutamate sources. In addition, the slope of the fit suggests that per mM change in [Glu] will lead to 1.31% change in the GluCEST contrast. This is in corroboration with the previous reports in various cerebral pathological conditions^3,4,9^.

We previously reported the elevation of GluCEST contrast and glutamate concentration measured via ^1^H MRS in the MPTP model of PD^9^. Histological data from that study showed increased GFAP immunoreactivity in the MPTP manifested as elevated astroglial reactivity and number, which has also been reported in prior work^21–23^. Another study found two-fold increase in glutamine synthetase (GS), attributed to a proliferation of glial cells^18^. The increase in GS and GFAP level may also point toward high level of glutamine which was not observed in the current study. This may be due to the early imbalance in the glutamate-glutamine neurotransmitter cycle reported previously. Further studies are required to determine the mechanism of elevated glial glutamate and GS and unaltered glutamine levels in the striatum followed acutely by MPTP exposure. Additionally, GFAP may not be used to measure the quantitative changes in the glial population followed by the neuroinflammation. There are other specific glial stains such as Crym gene, which is highly expressed by striatal astrocytes^24^. For the future studies, co-localization of anti-Glutamate and Crym stains may be performed for qualitative and quantitative analysis of striatal gliosis caused by MPTP.

However, in prior studies there was no compelling demonstration that the elevated glutamate was specifically derived from astrocytes. Both GluCEST MRI and ^1^H MRS measure the total glutamate in the brain which is mostly intracellular^25^. In the current study, confocal microscopy of double labeled brain slices showed cellular co-localization of glutamate and GFAP, demonstrating that the elevated Glu and GluCEST is primarily derived from intracellular glutamate in glial cells in the MPTP model.

## Conclusions

We confirmed the utility of GluCEST for detecting changes in striatal glutamate in the MPTP model of Parkinson’s Disease. GluCEST showed low intra- and inter-animal variability and demonstrated a strong association with changes in ^1^H MRS derived glutamate. Roughly 27% of GluCEST contrast appears to be derived from sources other than glutamate that are also not detectable by MRS, which agrees with the theoretical estimate from the previous study. Immunohistochemistry results confirmed cellular co-localization of glutamate and GFAP in the MPTP treated mouse brain, demonstrating that the elevated glutamate and GluCEST in the MPTP model is primarily from astrocytes.

## Methods

### MPTP administration in mice

The Institutional Animal Care and Use Committees (IACUC) of the University of Pennsylvania approved experimental protocols, and all experiments were carried out in accordance with approved IACUC guidelines. Male C57BL6 mice were procured from the Charles River Laboratory, Horsham, PA, USA and kept at the ULAR animal housing facility at the University of Pennsylvania. Mice were housed in a humidity controlled room ~22 °C under a 12-hour light/dark cycle with *ad libitum* access to food and water. Mice aged 3 months were divided into two groups: control (n = 16) and MPTP (n = 19). Mice in the MPTP group received MPTP (25 mg/kg, via intraperitoneal injection, Sigma/Aldrich, St. Louis, MO, USA) dissolved in normal saline once a day for 7 days, while the control mice received the same volume of normal saline for the same period.

### Acquisition and processing of ^1^H MRS data

All spectroscopy and imaging studies were performed on a 9.4 T horizontal 30 cm bore magnet fitted with a 12-cm gradient insert and interfaced to a Direct Drive MR spectrometer (Agilent Technologies Inc., Santa Clara, CA). Mice were anesthetized using 1.5–2% isoflurane mixed with O_2_ at 1 L/min, and secured to a body-bed inside a 20-mm diameter volume coil (M2M Imaging Corp., Cleveland, OH). Body temperature was monitored using a rectal temperature probe and maintained at 37 °C using warm air blown inside the bore of the magnet.

^1^H MR spectra were acquired from a voxel localized in the striatum (2 × 2 × 2 mm^3^) in control (n = 16) and MPTP treated (n = 19) mice using the Point RESolved Spectroscopy (PRESS) pulse sequence (TR/TE = 3000/18 ms, spectral width = 4 kHz, number of points = 4006, WET water suppression, averages = 256). The excitation pulse and refocusing pulses of bandwidth 8 kHz were offset to the glutamate resonance at 2.35 ppm (for minimization of the chemical shift displacement artifacts in calculating the glutamate concentration), while the water suppression pulse was offset at 4.7 ppm. Another spectrum was acquired without water suppression to obtain the water reference signal for normalization (averages = 8). Metabolite concentrations measured by *in vivo* ^1^H MRS were quantified using the LCModel software^12,26^. The concentration of metabolites was measured using the unsuppressed water peak as a concentration standard. Data were processed using LCModel, a least-squares based prior-knowledge fitting program. LCmodel applied a 9.4 T spin echo (TE = 25 ms) basis set incorporating the following resonances: alanine (Ala), aspartate (Asp), creatine (Cr), phosphocreatine (PCr), gamma-aminobutyric acid (GABA), glucose (Glc), glutamine (Gln), glutamate (Glu), glycerophosphocholine (GPC), phosphocholine (PCh), glutathione (GSH), myo-inositol (Ins), n-acetylaspartate (NAA), n-acetylaspartate + glutamate (NAAG), sycllo-inositol, taurine (Tau), lipid resonances at 0.9, 1.3 and 2.0 ppm and macromolecule resonances at 0.9, 1.2, 1.4, 1.7 and 2.0 ppm. A Cramer-Rao Lower Bound (CRLB) threshold of 10% was used to assure well-fitted data.

### Acquisition and processing of GluCEST MRI data

GluCEST, B_0_, and B_1_ maps were acquired from 1 coronal slice (2 mm thick) centered on the striatum. GluCEST images were acquired using a custom-programmed pulse sequence with a frequency selective saturation preparation pulse comprised of four square pulses, each with a duration of 250 ms with <10 μs delay between them (duty cycle 100%) at peak B_1_ of 250 Hz (5.87 µT) for the offset frequencies ±2.5, ±2.75, ±3, ±3.25, ±3.5 ppm from the water signal at 4.7 ppm, followed by a spoiled gradient echo (GRE) readout. The sequence parameters were as follows: field of view = 20 × 20 mm^2^, slice thickness = 2 mm, matrix size = 128 × 128, flip angle = 15°, GRE readout TR = 6.2 ms, TE = 2.9 ms, averages = 4, T_1_ recovery delay = 8 sec.

Segmentation of the striatum was performed manually on the CEST-weighted anatomical MR images, and ROIs were overlaid on the GluCEST maps. GluCEST contrast was measured as the asymmetry between an image obtained with saturation at the resonant frequency of exchangeable amine protons (+3 ppm downfield from water for glutamate), and an image with saturation equidistant upfield from water (−3 ppm), according to the following equation:

1 $$GluCES{T}_{asym({\rm{\Delta }}\omega =3ppm)}=\frac{{M}_{sat(-3ppm)}-{M}_{sat(3ppm)}}{{M}_{sat(-3ppm)}}\ast 100$$

where M_sat_ (±3 ppm) are the magnetizations obtained with saturation at Δω = ±3 ppm offset from the water resonance.

Radiofrequency field (B_1_) inhomogeneity and static magnetic field (B_0_) inhomogeneity in the brain slice were corrected using B_1_ and B_0_ maps generated from the same slice as described previously^1^. The B_0_ map was calculated by linearly fitting the accumulated phase per pixel following phase unwrapping against the echo time differences from GRE images collected at varying TE = 3.5, 4.0, and 4.5 ms. B_1_ maps were calculated from two images acquired using square preparation pulses with flip angles 30° and 60° (pulse duration = 65 μs, averages = 2) followed by a spoiled gradient echo. A flip angle map was generated, and a linear correction for B_1_ was calculated as a ratio of the actual B_1_ to the expected value.

In addition to the GluCEST scans, 3 animals from each group were used to perform MTR_asymm_ analysis. For this, all the acquisition parameters were the same as mentioned earlier except the saturation offsets were from 0 to ±5 ppm at the step size of 0.2 ppm.

### Reproducibility studies for GluCEST

A separate group of naive 2-month-old C57BL6 mice (n = 5) underwent repeated GluCEST MRI scans on 3 days with two days between scans to measure the intra-animal and inter-animal GluCEST variability. The coefficient of variation for inter-animal and inter-scan GluCEST measurements was calculated from the measured values.

### Background GluCEST contribution estimation

Tha background GluCEST contribution from the GluCEST and ^1^H MRS data was calculated using the information provided by plotting them on a scatter plot and finding the y intercept on the GluCEST axis.

The formula used for background contribution estimation is:

2 $$GluCES{T}_{Background}=\frac{GluCES{T}_{atGlu=0}}{GluCES{T}_{Average}}\times 100$$

### Immunohistochemical analysis of brain

A sub-set of mice MPTP treated (n = 5) and control PBS treated (n = 5) was prepared for immunohistochemistry (IHC). The mice were sacrificed using a standard method of transcardial perfusion/fixation with 15 ml of phosphate buffered saline (PBS) followed by 20 ml of 10% formalin. The brains were removed from the skull and stored overnight at 4 °C in 10% formalin. Paraffin-embedded sections were sliced at 6 μm thickness, and mounted on poly-lysine coated slides.

Glutamate (AB5018 Millipore) and GFAP (Z0334 Dako) antibodies were used to stain formalin-fixed, paraffin–embedded tissue. Paraffin was cleared with xylene and slides were rehydrated through descending concentrations of ethanol. Slides were then treated with 3% H_2_O_2_/methanol for 30 min. Sides were pretreated in a pressure cooker (Biocare Medical) in Antigen Unmasking solution (Vector Labs H3300). After cooling, slides were blocked in Sudan Black (199664-25 G, Sigma-Aldrich, St Louis, MO) for 20 min at RT. Slide were then rinsed in 0.1 M Tris Buffer then blocked with 2% fetal bovine serum for 15 min. Slides were then incubated with glutamate antibody at a 1:100 dilution for 1hr at room temperature. Slides were again rinsed then incubated with anti-rabbit polymer secondary pre-diluted (K4003, DAKO, Carpenteria, CA) for 30 min at room temp. After rinsing, slides were then incubated with the TSA biotin complex (NEL7490B001, Perkin Elmer) at 1:50 for 10 min at room temp. Slides were then rinsed and incubated with Alexa 488 Streptavidin secondary (A21370, Life Technologies, Eugene, OR) 1:200 for 30 min at room temp. After rinsing, slides were treated in preheated 5% SDS (CS-5585-28, Denville Scientific, Metuchen, NJ) for 7 min at 55 °C and then rinsed and blocked with 2% fetal bovine serum again before incubating with either anti-GFAP (Z0334, Dako) at 1:1 K or TH (NCL-TH Leica) at 1:50 for 1hr room temp. After rinsing, the slides were incubated for 1hr in Alexa 594 anti-rabbit or anti-mouse secondary respectively (A11012, Life Technologies, Eugene, OR). Slides were again rinsed and then counterstained with DAPI, and rinsed again before cover slipping with Prolong Gold (P36930, Life Technologies, Eugene, OR). After drying, the slides were scanned at 20 × magnification using and Aperio IF slide scanner (LeicaBiosystems).

### Confocal microscopy of brain slices

Confocal microscopy was used to examine the brain slices which had undergone single or double immunofluorescence for anti-glutamate and GFAP. Serial optical sections were collected (0.4 mm/step) on a confocal microscope (Leica DMIRBE). Each section was examined individually or was used to generate projections for either anti-glutamate (green, visualized with FITC-conjugated Igs), GFAP for astrocytes (red, visualized with Texas Red-conjugated Igs), or DAPI for nuclei labeling (blue). The three projections were merged to give simultaneous visualization of the three antigens. We ensured that co-localization, when apparent, was present on each section.

### Statistics

Statistical analyses were performed in JMP Pro 13 (SAS, Cary NC). The GluCEST and [Glu] values were taken from both the groups. The error in the GluCEST values was calculated using the standard error of average intra-scan GluCEST values while the [Glu] error was the SD values from the LCModel analysis.

We performed bivariate fit using 3 models to fit GluCEST with [Glu] with a p < 0.01 as significant. The most complex model assumed fitted GluCEST with [Glu], sample group i.e. Control or MPTP, and their cross term. Second model assumed different intercepts but same slopes for each sample group. The most parsimonious model fit a single linear fit for both groups. Residuals and normal quantile (Q-Q) plots of residuals were plotted for all models as a visual check of normal distribution.

## Electronic supplementary material

Supplementary Information
