# Supplementary material for: In vivo GluCEST MRI: Reproducibility, background contribution and source of glutamate changes in the MPTP model of Parkinson’s disease
Source: Sci Rep. 2018 Feb 13;8:2883. doi: 10.1038/s41598-018-21035-3 (PMC5811435; doi:10.1038/s41598-018-21035-3)
Supplement: Supplementary file 1 — Supplementary Information [file 41598_2018_21035_MOESM1_ESM.docx]

**In vivo GluCEST MRI: Reproducibility, background contribution and source of glutamate changes in the MPTP model of Parkinson’s disease**

Puneet Bagga^1^, Stephen Pickup^1^, Rachelle Crescenzi^1^, Daniel Martinez^2^, Arijitt Borthakur^1^, Kevin D’Aquilla^1^, Anup Singh^4^, Gaurav Verma^1^, John A. Detre^1,3^, Joel Greenberg^3^, Hari Hariharan^1^ and Ravinder Reddy ^1^

^1^ Center for Magnetic Resonance and Optical Imaging, Department of Radiology, University of Pennsylvania, Philadelphia, PA, United States

^2^ Department of Pathology and Laboratory Medicine, Children’s Hospital of Philadelphia, Philadelphia, Pennsylvania

^3^ Department of Neurology, University of Pennsylvania, Philadelphia, PA, United States

^4^ Centre for Biomedical Engineering, Indian institute of Technology, New Delhi, India

**Statistics**

The bivariate fit on the data was performed using 3 models to fit GluCEST with [Glu] and sample type with a p<0.01 as significant.

**Model 1**

The most complex model 1 fitted GluCEST with [Glu], sample type i.e. Control or MPTP, and their cross term. The results of fitting and residual plot are shown below.

**Summary of the fit:**

| RSquare | 0.89 |
| --- | --- |
| RSquare Adj | 0.88 |
| Root Mean Square Error | 0.66 |
| Mean of Response | 24.86 |
| Observations (or Sum Wgts) | 35.00 |

Excellent model. [Glu] can explain 89% of variance in GluCEST.

**Parameter Estimates:**

| **Term** | **Estimate** | **Std Error** | **t Ratio** | **Prob>\|t\|** |
| --- | --- | --- | --- | --- |
| Intercept | 9.34 | 1.65 | 5.65 | **<.001*** |
| [Glu] | 1.12 | 0.12 | 9.44 | **<.001*** |
| Sample type[Control]*([Glu]-13.7629) | -0.06 | 0.12 | -0.53 | 0.603 |
| Sample type[Control] | -0.38 | 0.15 | -2.47 | 0.019 |

Both slope and intercept are significantly different than zero (p<0.01) but sample type and cross term are not.

**Model 2**

Second model assumed different intercepts but same slopes for each sample group, i.e. fitted GluCEST with [Glu] and sample group i.e. Control or MPTP, and no cross term. The results of fitting and residual plot are shown below.

**Summary of the fit:**

| RSquare | 0.89 |
| --- | --- |
| RSquare Adj | 0.88 |
| Root Mean Square Error | 0.65 |
| Mean of Response | 24.86 |
| Observations (or Sum Wgts) | 35.00 |

Excellent model. [Glu] can explain 89% of variance in GluCEST %

**Parameter Estimates:**

| **Term** | **Estimate** | **Std Error** | **t Ratio** | **Prob>\|t\|** |
| --- | --- | --- | --- | --- |
| Intercept | 9.55 | 1.58 | 6.03 | **<.001*** |
| [Glu] | 1.11 | 0.12 | 9.61 | **<.001*** |
| Sample type[Control] | -0.38 | 0.15 | -2.53 | 0.016 |

Both slope and intercept are significantly different than zero (p<0.01) but sample type is not.

**Model 3**

The most parsimonious model fits a single linear fit for both groups. Residuals and normal quantile (Q-Q) plots of residuals were plotted for all models as a visual check of normal distribution.

Note: Dotted lines and gray shaded area around fit show 95% confidence interval region around fit.

**Summary of the fit:**

| RSquare | 0.86 |
| --- | --- |
| RSquare Adj | 0.86 |
| Root Mean Square Error | 0.70 |
| Mean of Response | 24.86 |
| Observations (or Sum Wgts) | 35.00 |

Excellent model. [Glu] can explain 86% of variance in GluCEST %

**Parameter Estimates:**

| **Term** | **Estimate** | **Std Error** | **t Ratio** | **Prob>\|t\|** |
| --- | --- | --- | --- | --- |
| Intercept | 6.81 | 1.25 | 5.45 | **<.001*** |
| [Glu] | 1.31 | 0.09 | 14.49 | **<.001*** |

Both slope and intercept are significantly different than zero (p<0.01).

**Residual fit** does not show any pattern to residuals indicating that normality assumption is valid.

**Normal Q-Q plot** further confirms normal distribution around fit.
